# Belowground interactions differ between sympatric desert shrubs under water stress

**DOI:** 10.1002/ece3.5999

**Published:** 2020-01-15

**Authors:** Zhengzhong Zhang, Lishan Shan, Yi Li, Yang Wang

**Affiliations:** ^1^ College of Forestry Gansu Agricultural University Lanzhou Gansu China

**Keywords:** plant–plant interactions, *Reaumuria soongorica*, root distribution, root morphology, *Salsola passerina*, stress gradient hypothesis

## Abstract

Understanding the relationships among species is central to ecological research; however, many knowledge gaps remain regarding how desert plant species interact. In the present study, we assessed the effect of rainfall on the belowground interactions and root morphology of two desert shrubs, *Reaumuria soongorica* (Tamaricaceae) and *Salsola passerina* (Chenopodiaceae), from three communities with similar landforms and soil environments. The roots of both *R. soongorica* and *S. passerina* were deeper when grown together than grown singly. Interestingly, the belowground biomass of *R. soongorica* was higher, but the belowground biomass of *S. passerina* was lower when grown together than when grown alone. This suggests that *S. passerina* benefitted from the association with *R. soongorica*. When grown together under conditions of low rainfall, the roots of *R. soongorica* were deeper than those of *S. passerina*, which suggests that *R. soongorica* is more robust than *S. passerina* when subjected to periods of decreased rainfall. We concluded that the symbiotic relationship between these two shrub species can lead to deeper roots and that the plants are affected by rainfall availability. Combined with the output results of climate change models, we speculated that the distribution area of these two species will expand to the west, which has important implications on how the interactions of other desert species may change in response to climate variability.

## Graphical abstract

The interspecific relationship of desert plants also has a significant impact on their morphological characteristics. Here, we found a novel interaction between two desert shrubs and proved that the effect will change with the environmental gradient.

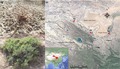

## 1 INTRODUCTION

Ecology is the study of the interactions among organisms and their environment. Most studies have focused on the relationship between organisms and the environment and, more specifically, the relationships between plants and animals. These studies have revealed many interesting phenomena, including coevolution (Baum, 2018; Ehrlich & Raven, 1964; Rausher, 2001), special signal transmission (Knauer & Schiestl, 2015), and parasitic relationships (Fritz, Moulia, & Newcombe, 1999; Schmidt et al., 2017; Su et al., 2017). Currently, there is a deficit in the information needed to classify the types of biological interactions. This may be because previous studies have mainly assessed the interactions between individuals and have neglected the effects of community‐level interspecies relationships. Additionally, previous studies have mostly focused on the interspecies relationships of plants at the species level (Adler et al., 2018; Mangla, Sheley, & Radosevich, 2011), and relatively, few studies have assessed the individual‐level relationships, especially in desert ecosystems.

**Figure 1:**
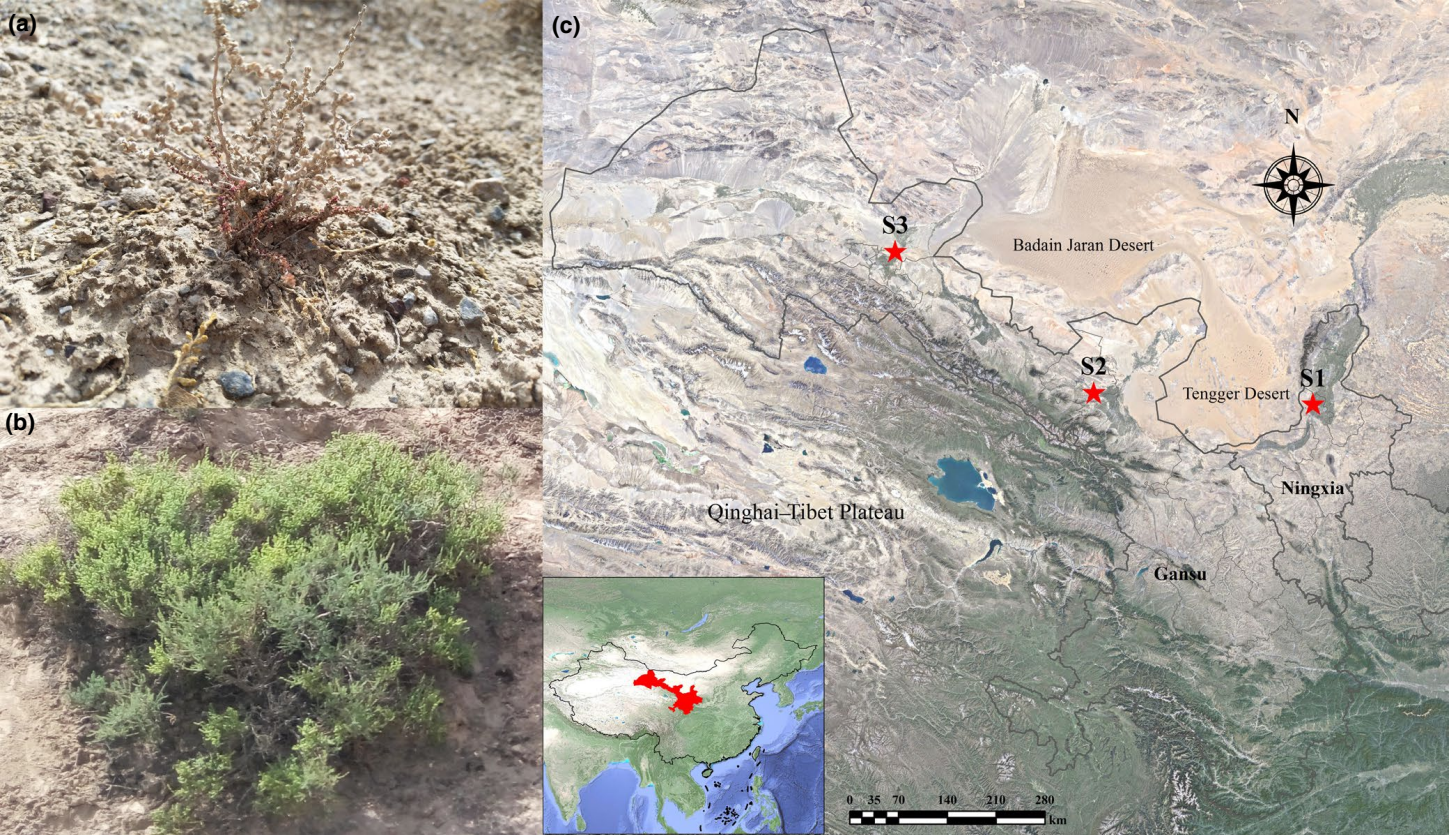
The paired growth pattern of *Reaumuria soongorica* and *Salsola passerina* and locations of the sample sites. Seedlings (a) and adult plants (b) of *R. soongorica* and *S. passerina* in the paired growth experiments. (c) Map of the sample site distribution in the Hexi Corridor; the insert indicates the main global codistribution areas of the two species. The sample sites S1, S2, and S3 represent moderate, suitable, and severe environments, respectively

*Reaumuria soongorica* and *Salsola passerina* are desert shrubs that are usually constructive or dominant species in the desert ecosystem of northwest of China (Chen, Zhu, Cao, Song, & Zhong, 2011; Zheng et al., 2008). Both shrubs exhibit two growth patterns, which is unusual among desert shrubs. In addition to growing independently, *Reaumuria soongorica* plants sometimes grow close (within a few centimeters or even less than a centimeter) to *S. passerina* plants, and their branches overlap each other and the roots become intertwined. Because the distribution of vegetation in deserts is generally sparse, this growth pattern does not usually occur between other desert species. In the present study, we referred to this growth pattern (i.e., of highly overlapping ecological niches) as “paired growth” (Figure 1a, b) and the widespread independent growth pattern of one plant species as “single growth.” These two growth patterns provide an ideal experimental basis for studying the interspecific relationship of desert plants.

Considering predicted global climate change scenarios, many studies have focused on the impact of environmental factors on the life cycles or the adaptability of animal and plant species; however, few studies have focused on how climate change might affect the relationships between plant species. Once the impact of climate change on species was demonstrated, it became clear that the disruption of the original balance between species would be inevitable.

The morphology of a plant root system can reflect adaptations to its environment (Stanley et al., 2018); however, most studies on plant root system morphology have mainly explored how plants acquire resources or adapt to climate change (Pilon et al., 2013). Some studies have examined the distribution of plant root systems within a single plant community, that is, looking at the relationships among species or individuals (Tron et al., 2015). The main influence of an interspecies relationship within the same vegetation type (except vines) is the distribution of roots among neighbors. In this study, we explored the differences between the growth patterns of the root systems of two desert shrubs.

In 1994, Bertness and Callaway proposed the stress gradient hypothesis (SGH), which stated that the competitive effect between plants decreases with an increase in environmental pressure and causes an increase in the promotion effect (Bertness & Callaway, 1994). This occurs because plants grown together can buffer harmful environmental stress on the individual level (Brooke et al., 2008). Most recent studies support the SGH (Choler, Michalet, & Callaway, 2001; Lortie & Callaway, 2006; Pugnaire & Luque, 2001; Ziffer‐Berger, Weisberg, Cablk, & Osem, 2014); however, some do not (Lamb, Kembel, & Cahill, 2009; Maestre, Callaway, Valladares, & Lortie, 2009). This discrepancy has stimulated the consideration of the effects of different environmental factors (Forey et al., 2010; He & Bertness, 2014; Smit, Rietkerk, & Wassen, 2009), measurement indicators (Chu et al., 2010; He, Bertness, & Altieri, 2013), research objects (Bertness & Yeh, 1994; Holzapfel & Mahall, 1999), and interactions (Smit et al., 2009). Moreover, many researchers have tried to modify the SGH. Few studies have assessed how changes in the environmental gradient, using rainfall as the dominant factor, affect the relationships between desert plant species.

Three hypotheses guided our study: (a) The primary environmental factor that affects the growth and distribution of *R. soongorica* and *S. passerina* is the quantity of annual rainfall, that is, plants at sites receiving greater rainfall would have a greater biomass. (b) The relationship between *R. soongorica* and *S. passerina* is mutualistic, that is, plants of both species would grow better when grown together than when grown singly. (c) The mutualism between *R. soongorica* and *S. passerina* is more intense in the presence of environmental stress, that is, that plant competition would be weakened and the positive interactions intensified by an increase in environmental stress (Bertness & Callaway, 1994). To tests these three hypotheses, we compared the growth of *R. soongorica*, and *S. passerina* grown singly and grown together under conditions with an environmental gradient. This study demonstrated the changes in the interactions between these two desert shrub species along a rainfall gradient and thus presents important indications of how desert species interactions may change in response to changing climatic conditions.

## 2 METHODS

### 2.1 Study area and site description

The main codistribution areas of *R. soongorica* and *S. passerina* occur in the northern Qinghai–Tibet Plateau, usually in the long and narrow area of the southern Badain Jaran and Tengger deserts (Figure 1c). This area has an arid or semi‐arid climate characterized by drought, low precipitation, high evaporation, and extreme temperature differences in summer (Li et al., 2017). We selected three sampling locations in a piedmont alluvial plain with thick soil layers that have low human impact (Figure 1c). Within each location, we selected sampling sites with similar landforms and soil environments that presented a gradient in climate and altitude (Table 1). The western part of S3 was sparsely distributed, and the eastern part of S1 was gradually replaced by other plant communities. The plant species structure of the sites was simple. In all sampling sites, *R. soongorica* and *S. passerina* were the dominant species; however, a few other species were present, for example, *Nitraria tangutorum*, *Lycium barbarum*, and *Kalidium foliatum*.

**Table 1:** Environmental factors of the three study areas in the Hexi Corridor, with moderate (S1), suitable (S2), and severe (S3) environments, respectively

Sample site	Altitude^a^ (m a.s.l.)	Latitude^a^	Longitude^a^	Annual rainfall^b^ (mm)	Annual average evaporation (mm)	Annual average temperature^b^ (°C)
S1	1,287	39°53′34″	98°47′22″	60	2,141	7.9
S2	1,538	38°10′39″	102°18′41″	165	2,020	8.0
S3	1,278	38°3′20″	106°15′19″	206	2,255	8.8

a Altitude, longitude, and latitude were measured in the field by GPS. m a.s.l: meters above sea level.

b Rainfall and temperature data are presented as the annual average values published by the local government departments.

### 2.2 Experimental design

#### 2.2.1 Growth status of existing vegetation

We surveyed the growth status of vegetation in each sampling site in October and September 2016. We randomly laid out three quadrats (100 × 100 m) that were situated at least 50 m away from each other. We further divided up each of the quadrats into plots (5 × 5 m). In each plot, we measured and recorded the plant height and crown breadth of all *R. soongorica* and *S. passerina*. We also counted how many of each species exhibited “paired growth” and “single growth” patterns. Density was measured as the total number of plants divided by the total area of the plot. The paired ratio was calculated as the number of paired growth patterns divided by the total number of plants.

$$\text{Paired ratio}=\frac{\text{Paired growth patterns}}{\text{Total plants}}\times 100\%$$

#### 2.2.2 Sample‐plant selection

In each study site, we sampled three representatives of single *R. soongorica*, single *S. passerina*, and paired *R. soongorica* and *S. passerina*. We specifically chose sample plants with normal growth forms that lacked signs of physical damage. To ensure the comparability and representativeness, the sampled plants were chosen between the highest distribution area of the plant height and crown breadth, based on the survey results.

#### 2.2.3 Sampling and processing

Height and canopy width were measured for single growing *R. soongorica* and *S. passerina* and for plants growing in close proximity. The aboveground parts were cut, placed in labeled ziplock bags, and refrigerated (0°C) to reduce the influence of respiration. The root systems were excavated (with a radius of 0.8 m from the base of each plant) with soil 10 cm belowground forming a layer until all of the root systems were dug out. Soils were screened with a 0.1 cm soil sieve, and stones and grit were removed. Roots were placed in labeled ziplock bags and refrigerated (0°C). All samples were transported to and stored in a laboratory. Roots of other plants were differentiated according to their color‐morphological texture flexibility, and *R. soongorica* and *S. passerina* roots were separated from each other. Soil adhering to the seedlings was brushed off, and the morphological index (e.g., root surface area) of the root systems was analyzed using Epson scanners and WinRHIZO Basic 2008a. Roots and stems were separately dried in an oven at 105°C for 2 hr and then maintained at 80°C for 48 hr, after which the dry biomass (aboveground biomass and each layer of belowground biomass) was determined using an analytical balance.

#### 2.2.4 Calculation

The root extinction coefficients of *R. soongorica* and *S. passerina* under conditions of single growth and paired growth were calculated using Gale's nonlinear model on plant root vertical distribution (Gale & Grigal, 1987), as follows:

$$Y=1-\beta^{d}$$

where *Y* represents the cumulative percentage of root biomass from the soil surface to the depth *d* (cm) of a certain soil layer, and *β* is the root extinction coefficient. The larger the value of *β* (closer to 1), the greater the proportion of plant roots distributed in deeper soil layers.

The root biomass paired‐single ratio represents the mean paired root biomass of a species to the mean single root biomass and was calculated as follows:

$$\text{Paired ratio}=\frac{\text{Paired growth patterns}}{\text{Total plants}}\times 100\%$$

where a ratio larger than 1 indicates that the root system was more developed in paired growth than single growth, and a ratio smaller than 1 indicates that the root system was more developed in single growth than paired growth.

#### 2.2.5 Data use and statistical analysis

We measured the effect of paired versus single growth by comparing the vertical distribution of roots of paired and independently (single) growing plants. The root extinction coefficients of all plants were curve‐fitted by MATLAB9.0. The growth patterns and the index change with environmental gradients were compared. Data that met the assumptions of normality and homogeneity were analyzed by two‐way ANOVA and Student–Newman–Keuls tests in SPSS21.0. Other data were tested using a nonparametric analysis and Wilcoxon rank‐sum test. Significant differences were determined based on multiple comparisons using Kruskal–Wallis tests. The changes in the paired growth of the two plant species in response to varying environmental gradients were then compared.

## 3 RESULTS

**Figure 2:**
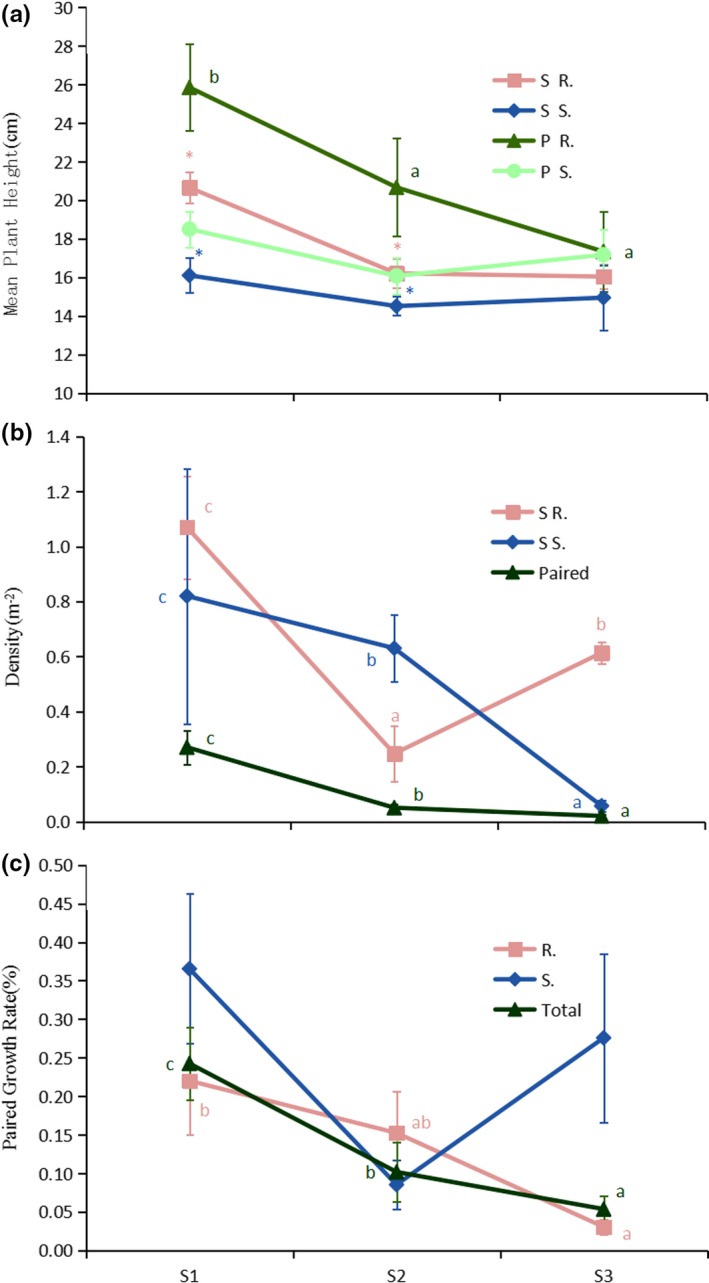
*Reaumuria soongorica* and *Salsola passerina* growth conditions at the three sample locations: moderate (S1), suitable (S2), and severe (S3) environments. (a) Mean plant height. (b) Plant density, calculated as the mean number of plants per m^2^. (c) The paired ratio, calculated as the number of paired growth patterns divided by the total number of plants. S = single growth; P = paired growth; S. = *S. passerina*; R. = *R. soongorica.* Asterisks (*) indicate significant differences (*p* < .05) between the two growth patterns. Different lowercase letters indicate significant differences (*p* < .05) between sites

Plant communities dominated by *R. soongorica* and *S. passerina* were sparsely distributed. The height of *R. soongorica* plants decreased significantly with the increase in environmental gradients (Table 2), whereas the height of *S. passerina* plants was not significant affected by the change in environmental gradients (Figure 2a). In addition, the height of both plant species was higher when grown under paired conditions than when grown singly (Figure 2a). The density (Figure 2b) and paired growth rate (Figure 2c) generally declined with the increase in the environmental gradient (i.e., as the conditions became harsher). However, in S3 (the most severe environments), the density of *R. soongorica* and the paired rate of *S. passerina* were higher than in S2 (suitable environments).

**Table 2:** The mean plant height, density, and paired ratio of *Reaumuria soongorica* and *Salsola passerina* growing in moderate (S1), suitable (S2), and severe (S3) environments in the Hexi Corridor

	S1	S2	S3
Mean plant height (cm)			
S R.	20.65 ± 0.79b*	16.20 ± 0.77a*	16.03 ± 0.63a
S S.	16.10 ± 0.90a*	14.50 ± 0.49a*	14.94 ± 1.69a
P R.	25.85 ± 2.24a	20.67 ± 2.54a	17.33 ± 2.09a
P S.	18.50 ± 0.93a	16.07 ± 0.96a	17.17 ± 1.30a
Density (m^2^)			
S R.	1.07 ± 0.19c	0.25 ± 0.10a	0.61 ± 0.04b
S S.	0.82 ± 0.46b	0.63 ± 0.12b	0.06 ± 0.02a
Paired	0.27 ± 0.06b	0.05 ± 0.01a	0.02 ± 0.01a
Paired ratio (%)			
S R.	0.22 ± 0.07b	0.15 ± 0.05ab	0.03 ± 0.01a
S S.	0.37 ± 0.10a	0.09 ± 0.03a	0.28 ± 0.11a
Total	0.24 ± 0.05b	0.10 ± 0.04b	0.05 ± 0.02a

Note

S = single growth; P = paired growth; S. = *S. passerina*; R. = *R. soongorica*. Asterisks (*) indicate significant differences (*p* < .05) between the two growth patterns. Different lowercase letters indicate significant differences (*p* < .05) between sites.

**Figure 3:**
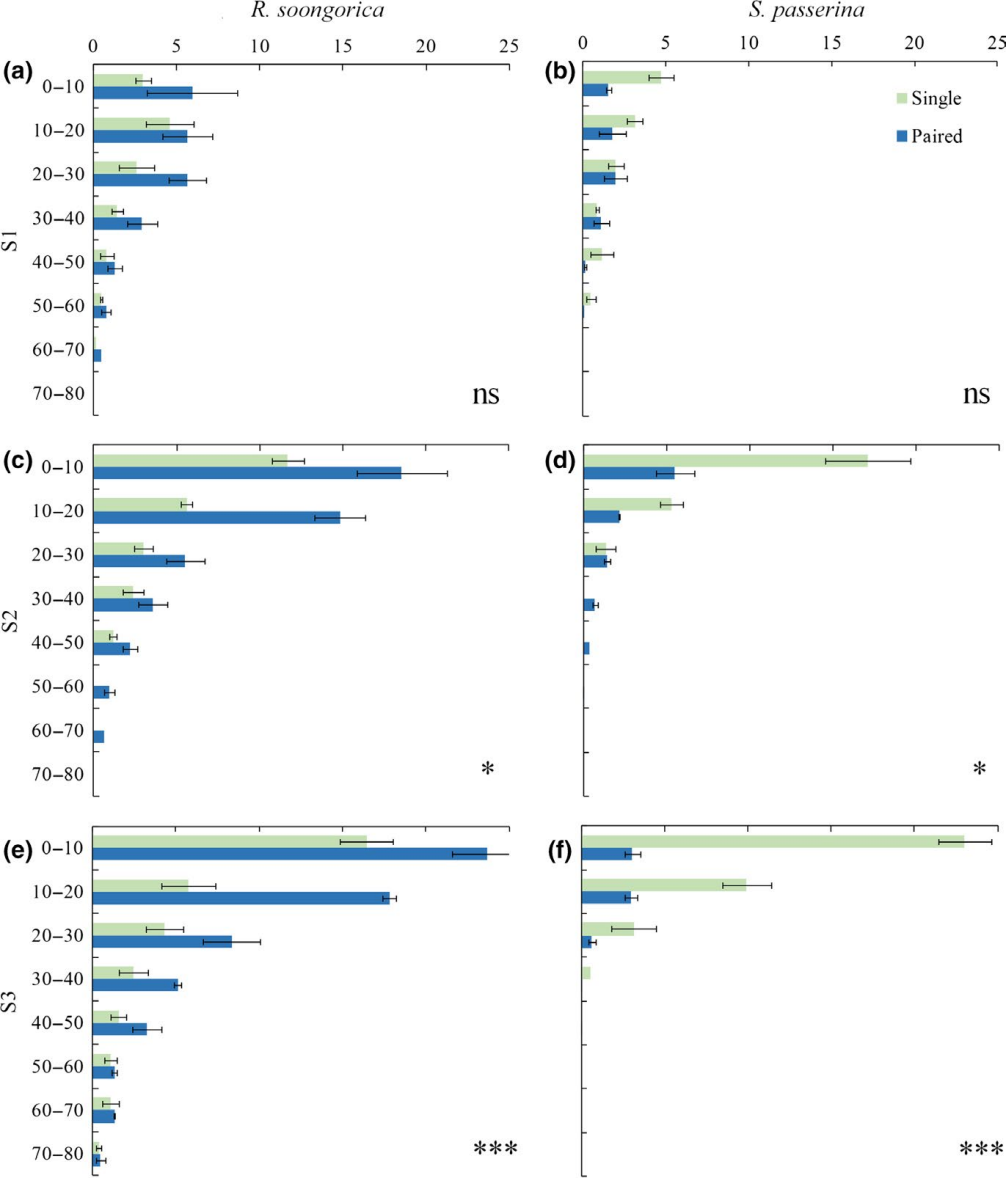
The vertical distribution of root biomass (g) of *Reaumuria soongorica* (a, c, e) and *Salsola passerina* (b, d, f) with two growth styles (paired and single) in different environments: S1 moderate (a, b); S2, suitable (c, d); and S3, severe (e, f). Green indicates single growth, and blue indicates paired growth. Significant differences in the total root biomass between the two growth treatments: *p < .05, **p < .01, ***p < .001; ns, not significant at *p* > .05. Bars and error bars represent means ± *SE*s (*n* = 3)

The results of ANOVA revealed that the underground biomass of both *R. soongorica* and *S. passerina* was significantly (*p* < .05) smaller in sites with lower annual rainfall (Table 3). When grown alone, *R. soongorica* commonly formed a tap root with a deeper root system. In contrast, *S. passerina* grown alone had fibrous root systems and roots that were mainly distributed in shallow layers. Compared with single growing plants, the root biomass of *R. soongorica* in all soil layers was higher when grown with *S. passerina*. In contrast, *S. passerina* root biomass was significantly (*p* < .01) lower when grown with *R. soongorica* than when grown alone. In addition, roots of *S. passerina* grew deeper, and the biomass of its root system in the lower soil layers increased. In both species, the difference between the growth in the paired‐ and single growth treatments increased with an increase in the severity of the environments (Figure 3).

**Table 3:** The root surface area and biomass of *Reaumuria soongorica* and *Salsola passerina* growing in moderate (S1), suitable (S2), and severe (S3) environments in the Hexi Corridor

	*R. soongorica*		*S. passerina*	
	Single	Paired	Single	Paired
Surface area				
S1	401.74 ± 30.83b*	1,500.17 ± 84.11a	1683.87 ± 112.77a*	981.40 ± 126.90a
S2	514.55 ± 26.69b*	1,325.72 ± 106.62a	816.27 ± 111.29b	827.50 ± 161.60a
S3	1,100.21 ± 23.93a*	1648.67 ± 56.70a	748.60 ± 63.55b**	538.92 ± 60.48a
Biomass				
S1	12.91 ± 1.91c*	22.27 ± 2.97c	11.80 ± 1.09c*	6.59 ± 1.73a
S2	23.81 ± 2.18b*	45.78 ± 4.62b	23.74 ± 2.83b*	10.05 ± 1.46a
S3	33.14 ± 1.79a*	61.73 ± 1.33a	36.28 ± 1.91a*	6.73 ± 0.97a

Note

Asterisks (*) indicate significant differences between the two growth patterns. Different lowercase letters indicate significant differences (*p* < .05) within sites.

**Figure 4:**
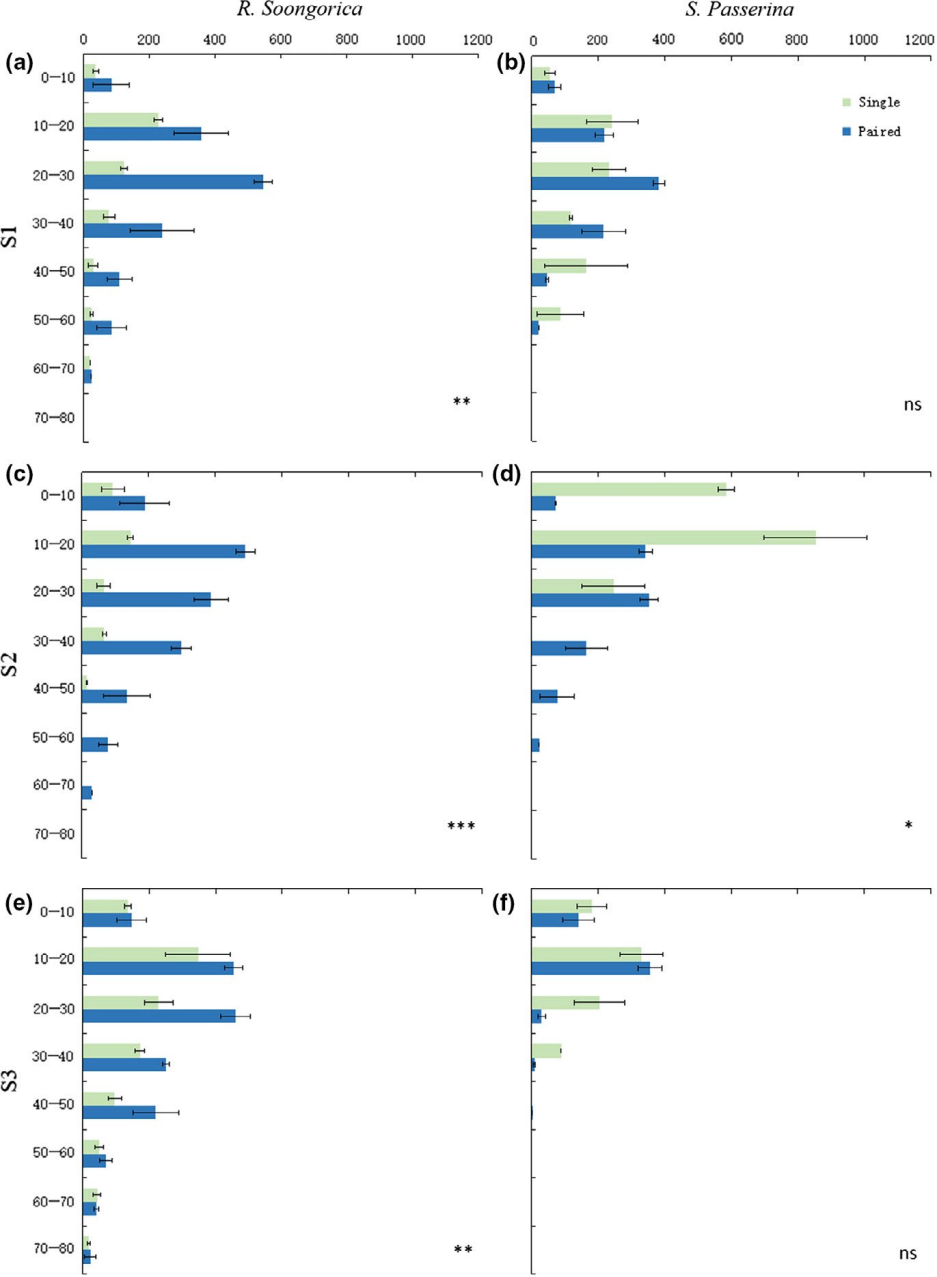
The vertical distribution of root surface area (cm^2^) of *Reaumuria soongorica* (a, c, e) and *Salsola passerina* (b, d, f) with two growth styles (paired growth and single growth) in different environments: S1 moderate (a, b); S2, suitable (c, d); and S3, severe (e, f)*.* Green indicates single growth, and blue indicates paired growth. Significant differences between the two growth styles (*t* test): *p < .05, **p < .01, ***p < .001; not significant (ns) at *p* > .05. Bars and error bars represent means ± *SE*s (*n* = 3)

In all sample areas, the root surface area of *R. soongorica* was significantly (*p* < .01) larger in the paired growth conditions than when grown alone, whereas the difference between paired and singly grown *S. passerina* was smaller. The vertical distribution of *R. soongorica* root systems among singly grown and paired plants was similar; furthermore, the vertical distribution of the root surface area was similar between *S. passerina* and *R. soongorica*. The root surface area was mainly distributed in the top 10–40 cm of soil. In suitable environments (S2), the growth of paired *S. passerina* plants was similar to the growth of singly growing plants; however, paired plants had deeper root systems: the surface area of singly grown plants was mainly concentrated in the top 0–20 cm and the 10–30 cm layer in paired plants (Figure 4).

**Figure 5:**
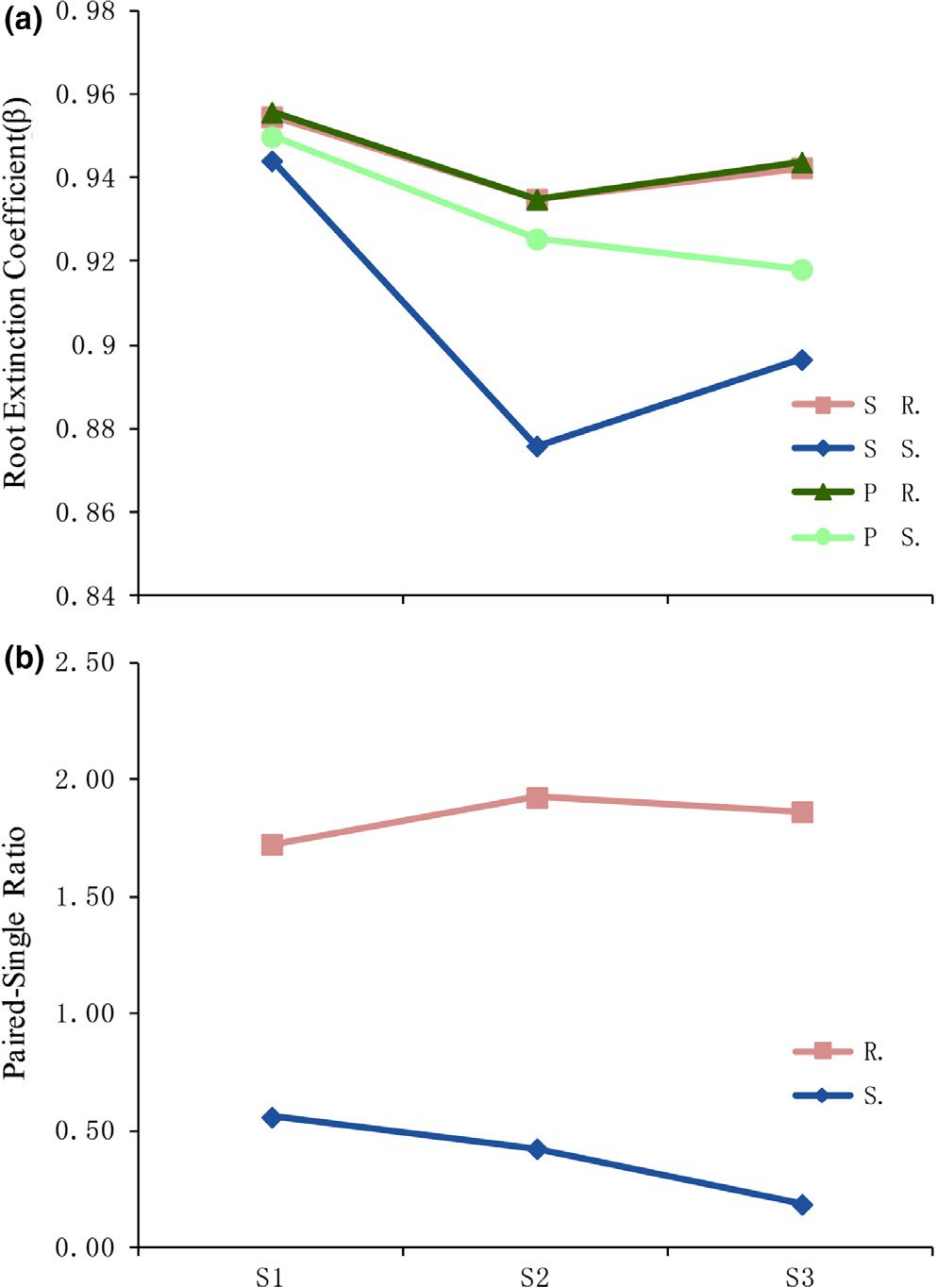
Effect of environments and growth treatment (paired or single growth) on the root systems of *Reaumuria soongorica* and *Salsola passerina*. (a) The root extinction coefficients (*β*) of *R. soongorica* and *S. passerina* in different environments. (b) Paired‐single ratio of root total biomass. Environments: moderate (S1), suitable (S2), and severe (S3). Bars and error bars represent means ± *SE*s (*n* = 3)

The root extinction coefficients of *R. soongorica* were similar for all plants (paired and singly grown) and were not significantly (*p* > .05) affected by the environmental gradient. The root extinction coefficients of paired *S. passerina* plants were higher (i.e., roots were deeper) than singly grown plants, when grown in a suitable (i.e., S2) environment (Figure 5a). The root biomass paired‐single ratio of *R. soongorica* was greater than 1. The root biomass paired‐single ratio of *S. passerina* was <1 and decreased with the increase in the environmental gradient (Figure 5b).

## 4 DISCUSSION

Our findings supported the first hypothesis, that annual precipitation is the primary environmental factor that affects the growth and distribution of *R. soongorica* and *S. passerina*. By choosing sites with similar latitudes, topographies, and soils, we were able to test for a potential correlation between the growth and distribution of the two study species in response to altitude and rainfall. Only plant density and annual rainfall were significantly negatively correlated (*p* < .05), and no significant correlations were observed between the plant growth attributes and environmental factors tested. In arid regions, moisture is the primary limiting factor (Zhang, Zhao, Liu, Fang, & Feng, 2016) and rainfall is the main source of plant water (Nie, Chen, Wang, & Yang, 2012). Thus, according to the “most limiting factor” concept presented by Liebig (1840), moisture is the primary limiting factor for plants in arid ecosystems (Thomas, Hoon, & Dougill, 2011). The decreased distribution of the two plants in the eastern sites with larger annual rainfall may, therefore, be the result of competition with other plants (Maron, Laney Smith, Ortega, Pearson, & Callaway, 2016).

Our findings only partially supported the second hypothesis, that paired growth conditions are not beneficial for both *S. passerina* and *R. soongorica*. The biomass of *S. passerina* root systems was lower when grown with *R. soongorica* than when grown alone. In suitable environments, paired conditions also resulted in *S. passerina* roots growing deeper. The root system of *S. passerina* is fibrous under single growth conditions, and the biomass and root surface area of the roots were found to be concentrated near the surface, indicating that rainfall was the main source of water for plants growing singly. In contrast, paired *S. passerina* plants characteristically had a taproot system (similar to *R. soongorica*, Figure 5a), in which the root surface area of the main distribution layer was in the 0–20 cm layer for paired plants and in the 10–30 cm layer for single plants (Figure 4c, d). This indicated that under paired growth *S. passerina* benefited from the water retention and hydraulic lift of the *R. soongorica* root system and was able to use the water retained in the soil. *Salsola passerina* benefited from growing with *R. soongorica*, and the root biomass of *S. passerina* in each soil layer was greater when grown with *R. soongorica* (paired conditions) than when grown alone. There are many potential causes for the increased underground biomass of plants, such as elevated CO_2_ levels, increases in temperature and rainfall (Kardol et al., 2010), decreases in rainfall (Fiala, Tůma, & Holub, 2009), and the effect of neighboring plants (Hendriks et al., 2015). Both the increase and decrease in rainfall may increase root biomass, whereby plants grow well and have larger plant parts under favorable conditions; however, root morphologies may not necessarily change substantially. However, under water stress, plants need to distribute more biomass underground to expand their roots to acquire more water. In this study, the aboveground parts of the same sampling sites were similar sized, and the significant increase in the underground parts represented a significant increase in the ratio of the root system. We speculated that the acquisition of water by *S. passerina* resulted in *R. soongorica* suffering water stress. *Reaumuria soongorica* therefore allocated more biomass to expand its root system. In summary, when *R. soongorica* and *S. passerina* grow in close proximity, *R. soongorica* is negatively impacted, but *S. passerina* benefits. The relationship between these two species can be summarized as interspecific competition. In addition, the plant heights of *R. soongorica* and *S. passerina* at each sampling point were higher when grown together than when grown alone, which may be a result of the competition for limited canopy space resources.

Our third hypothesis was not supported. With the increased environmental pressure (water stress), only the root biomass and its difference between two species were significant (Figure 3); the other index had no discernable trend. This finding may help to explain why the mean plant height and plant density decreased with the increased environmental gradient in the paired condition. Under conditions of high environmental stress (e.g., at S3), *R. soongorica* was negatively impacted by the paired growth (i.e., had a significantly larger root biomass, Figure 3e); however, *S. passerina* received some benefit from the paired growth (i.e., had an extremely significantly lower root biomass, *p* < .001, Figure 3f). In the SGH proposed by Bertness and Callaway (1994), increased environmental stress leads to increased intensity and the importance of mutual benefits. The SGH has been confirmed by many studies (Mori, 2018), but some studies have shown that this hypothesis is not always tenable (Lett, Wardle, Nilsson, Teuber, & Dorrepaal, 2017). Under extreme stress conditions, facilitation can disappear or become competition. To a certain extent, the SGH may explain the results of our study (O'Brien, Pugnaire, Armas, Rodríguez‐Echeverría, & Schöb, 2017; Wright, Schnitzer, & Reich, 2015). However, we only assessed one type of interaction (root morphology), and thus, our results may not be generalizable. As a field observation study, this study had some uncontrolled factors. In the future, it is necessary to perform controlled experiments in which more variables can be controlled to allow for the exploration of factors other than the root system. Such experiments will further our understanding of the stability and adaptability of *R. soongorica* and *S. passerina* and the interspecific relationships between them.

In this study, precipitation was the limiting factor. Our findings have important implications given that the global climate change model predicts that precipitation will continue to rise in the region for decades to come (IPCC, 2014). Additional observational studies may increase the accuracy of predictions regarding the future distribution of desert shrubs such as *R. soongorica* and *S. passerina.* For example, these drought‐tolerant shrubs may move westwards as a result of increased precipitation. The distribution and density of each species may also change in response to climate variations, and in some cases, their ranges may even increase (Kolanowska et al., 2017).

## CONFLICT OF INTEREST

The authors declare there are no conflicts of interest.

## AUTHOR CONTRIBUTIONS

LS and YL conceived and designed the experiment; ZZ and YW performed the experiment and collected the data; ZZ analyzed the data and wrote the first draft of the manuscript; and all authors contributed substantially to the revisions.

## Data Availability

All data that support this article are accessible in the Dryad repository: https://doi.org/10.5061/dryad.g1jwstqmz
